# Supplementary material for: Avian raptors are indicator species and victims of high pathogenicity avian influenza virus HPAIV H5N1 (clade 2.3.4.4b) in Germany
Source: Sci Rep. 2024 Nov 20;14:28779. doi: 10.1038/s41598-024-79930-x (PMC11579473; doi:10.1038/s41598-024-79930-x)
Supplement: Supplementary file 2 — Supplementary Material 2 [file 41598_2024_79930_MOESM2_ESM.pdf]

Figure legend of Supplemental Figure 1a, 1b and 1c

Median-joining network for concatenated whole genome HPAIV H5 sequences of genotypes (1a) Ger-10-21-N1.2 (corresponding to European genotype "C"), (1b) Ger-10-21-N1.5 (corresponding to European genotype "AB") and (1c) Ger-10-21-N1.5 (corresponding to European genotype "AB"). Sequences are represented by circles, colored as indicated in the legend according to their host group of origin. The diameter of the circle indicates the number of identical sequences represented. Vertical dashes along the network edges reflect mutational steps separating the sequences. Uncolored nodes refer to unsampled and therefore unknown intermediate sequences as estimated by the clustering algorithm. Clusters with at least one raptor sample (red dot) are highlighted in grey and labelled with consecutive Roman numbers. HPAIV H5 sequences from raptor species are labelled with the name of the host species (as provided), the year of sample collection and the EpiFlu accession number. The data underlying the figure are summarized in Supplementary Tables 5 and 6.

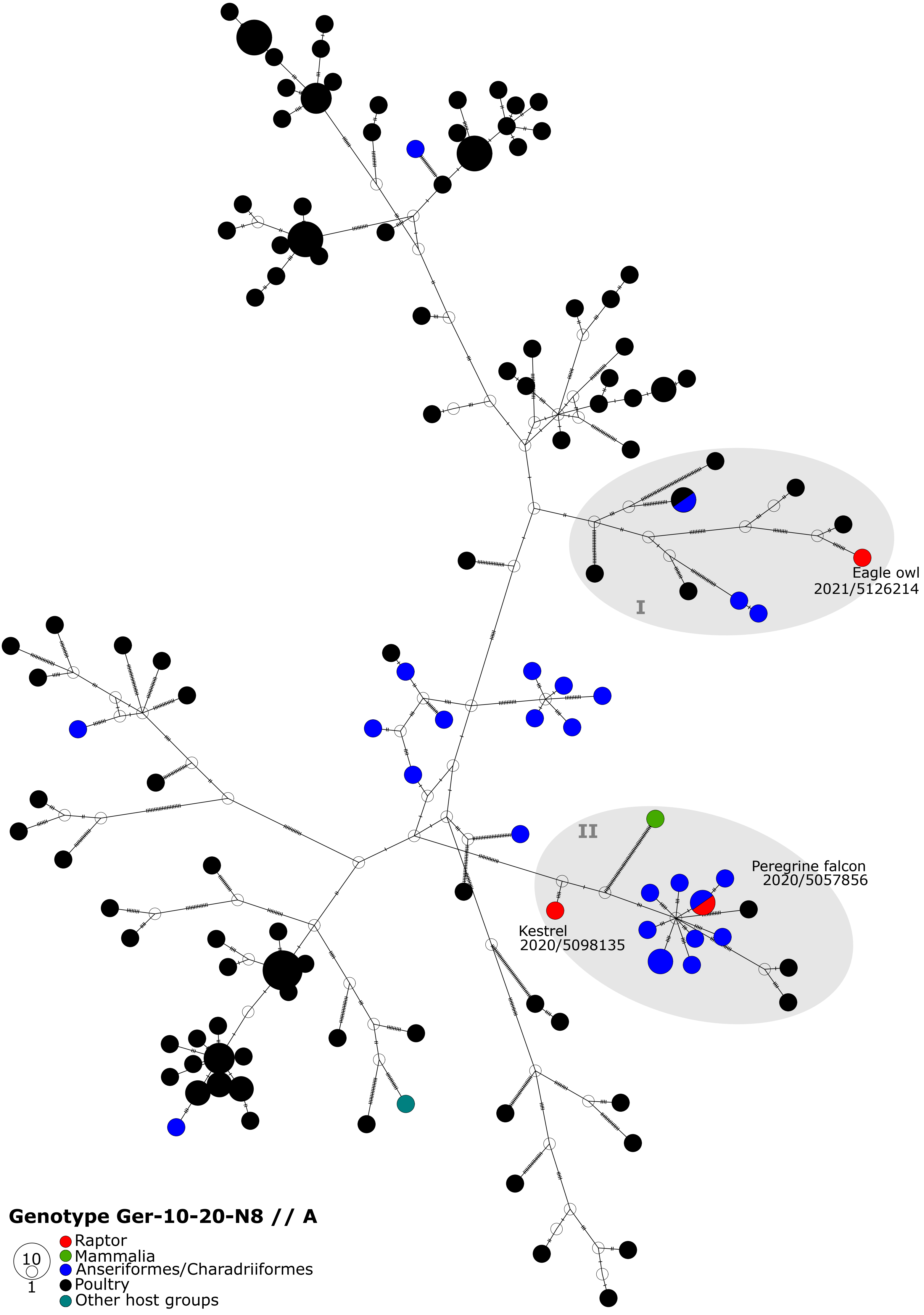
Eagle owl
2021/5126214
I
II
Peregrine falcon
2020/5057856
Kestrel
2020/5098135
Genotype Ger-10-20-N8 // A
10
1
Raptor
Mammalia
Anseriformes/Charadriiformes
Poultry
Other host groups

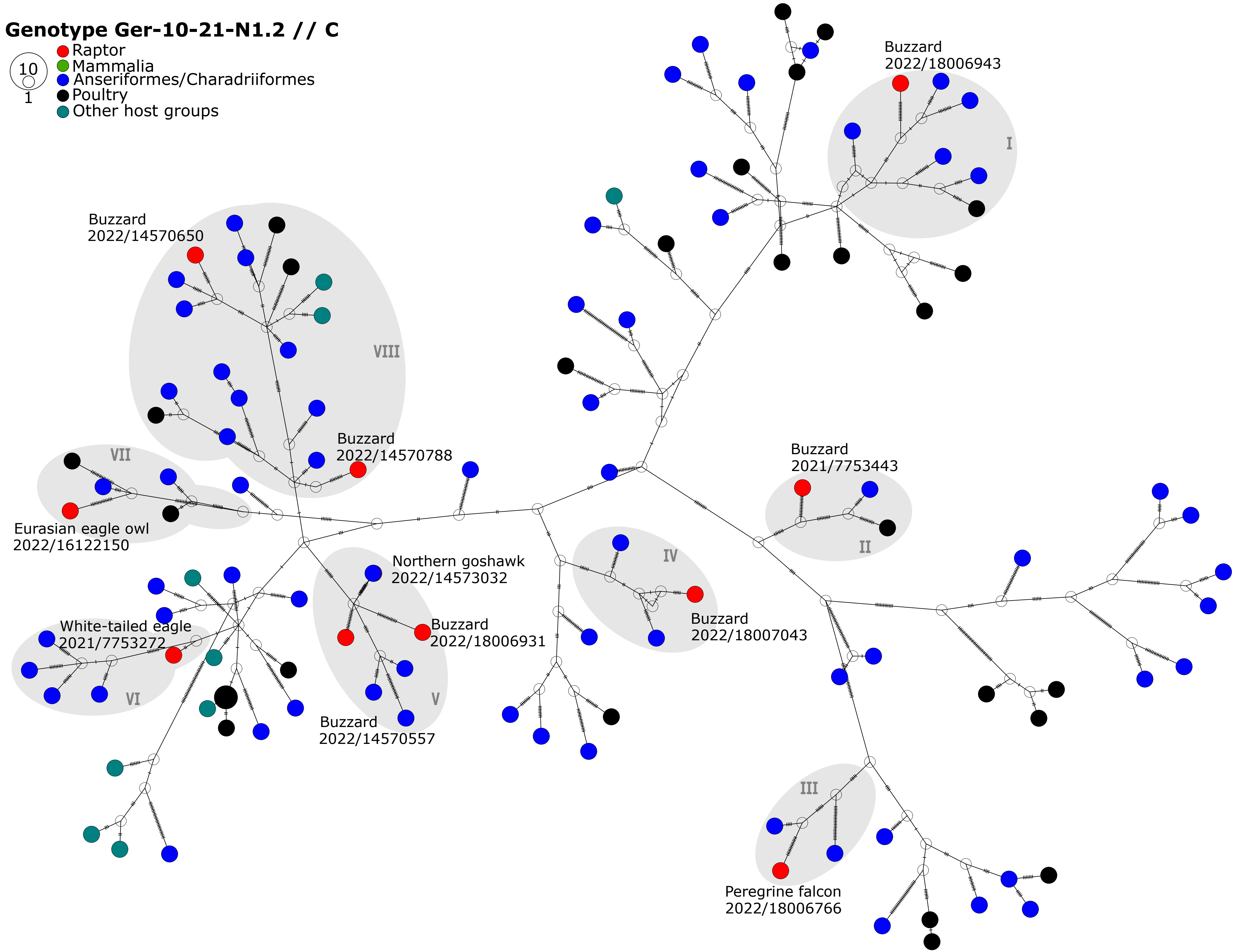
Genotype Ger-10-21-N1.2 // C
10
1
Raptor
Mammalia
Anseriformes/Charadriiformes
Poultry
Other host groups
Buzzard 2022/18006943
I
Buzzard 2022/14570650
VIII
Buzzard 2022/14570788
VII
Eurasian eagle owl 2022/16122150
Buzzard 2021/7753443
II
Northern goshawk 2022/14573032
IV
White-tailed eagle 2021/7753272
Buzzard 2022/18006931
Buzzard 2022/18007043
VI
V
Buzzard 2022/14570557
III
Peregrine falcon 2022/18006766

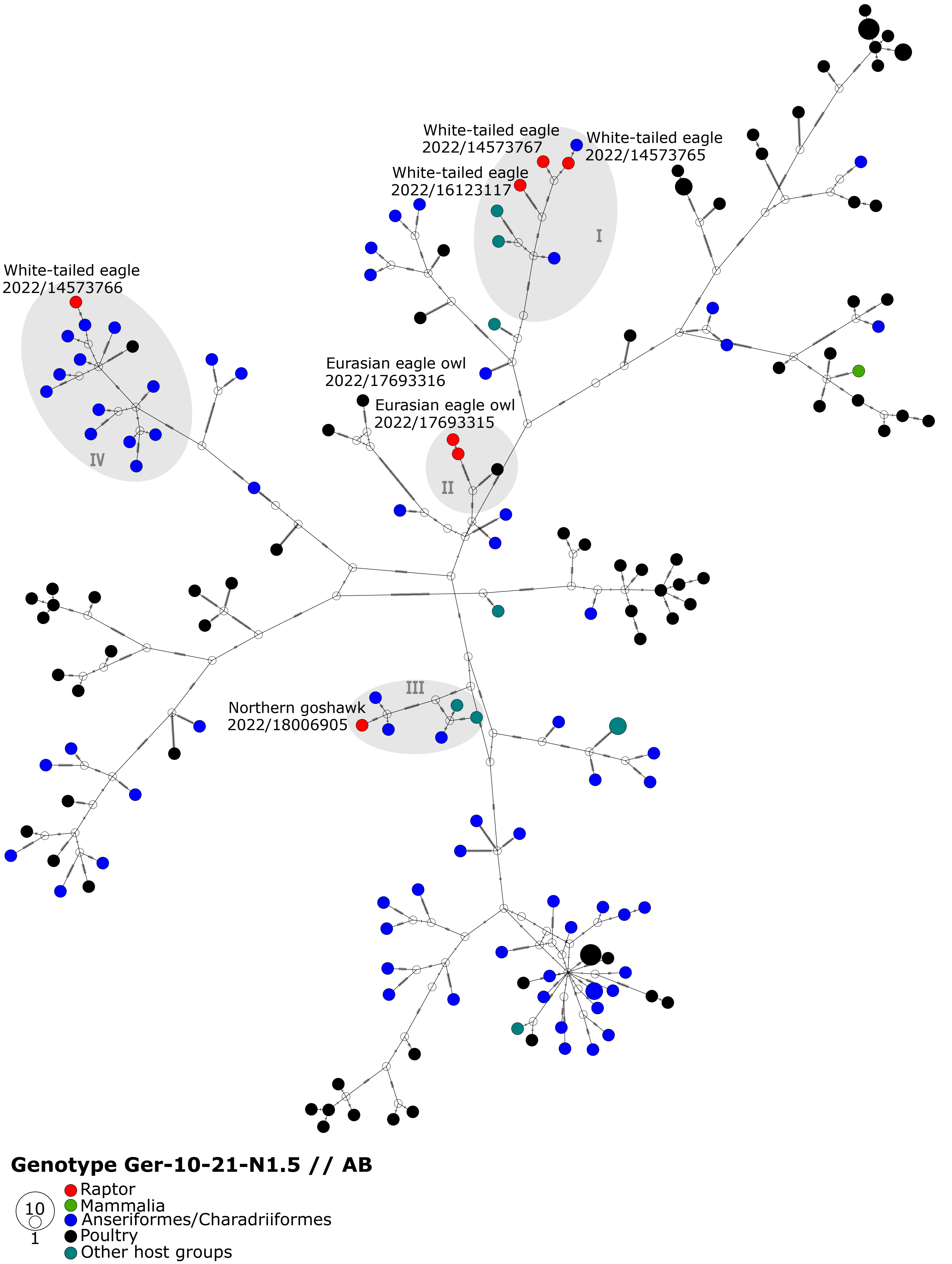
White-tailed eagle
2022/14573767
White-tailed eagle
2022/14573765
White-tailed eagle
2022/16123117
I
White-tailed eagle
2022/14573766
IV
Eurasian eagle owl
2022/17693316
Eurasian eagle owl
2022/17693315
II
III
Northern goshawk
2022/18006905
Genotype Ger-10-21-N1.5 // AB
10
1
Raptor
Mammalia
Anseriformes/Charadriiformes
Poultry
Other host groups
